# Mer receptor tyrosine kinase mediates both tethering and phagocytosis of apoptotic cells

**DOI:** 10.1038/cddis.2015.18

**Published:** 2015-02-19

**Authors:** I Dransfield, A Zagórska, E D Lew, K Michail, G Lemke

**Affiliations:** 1 MRC Centre for Inflammation Research, Queen's Medical Research Institute, University of Edinburgh, Edinburgh, UK; 2 Molecular Neurobiology Laboratory, The Salk Institute for Biological Studies, 10010 N. Torrey Pines Rd., La Jolla, CA, USA

## Abstract

Billions of inflammatory leukocytes die and are phagocytically cleared each day. This regular renewal facilitates the normal termination of inflammatory responses, suppressing pro-inflammatory mediators and inducing their anti-inflammatory counterparts. Here we investigate the role of the receptor tyrosine kinase (RTK) Mer and its ligands Protein S and Gas6 in the initial recognition and capture of apoptotic cells (ACs) by macrophages. We demonstrate extremely rapid binding kinetics of both ligands to phosphatidylserine (PtdSer)-displaying ACs, and show that ACs can be co-opsonized with multiple PtdSer opsonins. We further show that macrophage phagocytosis of ACs opsonized with Mer ligands can occur independently of a requirement for *α*V integrins. Finally, we demonstrate a novel role for Mer in the tethering of ACs to the macrophage surface, and show that Mer-mediated tethering and subsequent AC engulfment can be distinguished by their requirement for Mer kinase activity. Our results identify Mer as a receptor uniquely capable of both tethering ACs to the macrophage surface and driving their subsequent internalization.

Many diseases, including rheumatoid arthritis, pulmonary fibrosis, adult respiratory distress syndrome, and inflammatory bowel disease,^1, 2, 3, 4^ are commonly marked by impaired resolution of inflammation that is linked to defects in the phagocytic clearance of apoptotic cells.^5, 6, 7^ Apoptotic cell (AC) clearance normally eliminates a plethora of pro-inflammatory stimuli,^8, 9^ and the recognition of ACs by phagocytes^10^ limits progression to necrosis,^11^ suppresses pro-inflammatory mediator production, and induces IL-10 and TGF-*β* release.^12, 13^ As defective clearance of ACs is associated with the development of inflammatory disease and autoimmunity,^14, 15^ new therapeutic approaches designed to increase the capacity of phagocytes to remove ACs could effectively promote the resolution of inflammation.

Phagocytosis of ACs can be regulated by soluble mediators, including cytokines,^16, 17^ prostaglandins and lipoxins,^17, 18, 19^ serum proteins,^20^ agonists of Liver X receptors (LXRs),^17, 21^ and glucocorticoids (GC).^17, 22^ In particular, LXR agonists and GCs promote phagocytosis of ACs predominantly via a Tyro3/Axl/Mer (TAM) receptor tyrosine kinase (RTK)-dependent pathway.^17, 21, 23^ There are two established ligands for the TAM RTKs, Protein S (gene name *Pros1*), which activates Tyro3 and Mer, and Gas6, which activates all three TAMs,^24, 25^ although other ligands have been suggested.^26, 27^ The amino terminal Gla domains of Protein S and Gas6 bind to phosphatidylserine (PtdSer) on the plasma membrane of ACs,^28^ a potent ‘eat-me' signal by which ACs are recognized by phagocytes.^29^ TAM receptors bind to the carboxy terminal domains of Protein S and Gas6, which effectively act as molecular ‘bridges' between PtdSer on the AC and TAM receptors on the phagocyte.^17, 30, 31^ TAM receptor- and ligand-deficient mice exhibit defective phagocytic pruning of photoreceptor outer segments by retinal pigment epithelial (RPE) cells of the eye,^32, 33, 34^ defective clearance of apoptotic germ cells by Sertoli cells of the testis,^35^ and defective clearance of ACs by macrophages/dendritic cells in lymphoid organs.^36^ These phenotypes are also detectable in Mer (gene name *Mertk*) single knockouts.^37^ In addition to phagocytic clearance, TAM signaling also has a pivotal role in controlling the innate immune response to pathogenic stimuli.^13, 17, 38^

Although the importance of Mer in the internalization of ACs by macrophages is now well-established, this receptor has been thought not to have a significant role in the initial ‘tethering' of ACs to the macrophage surface.^36, 39^ In their studies, Scott *et al.*^36^ used peritoneal macrophages for which tethering of ACs has now been shown to be mediated by T-cell immunoglobulin and mucin domain-containing molecule 4 (TIM4).^39^ Subsequent internalization of tethered ACs is then mediated by either integrin *α*v*β*3- or Mer-mediated signaling.^39, 40^ Similarly, for RPE cells, the initial capture of photoreceptor outer segments by RPE cells required the integrin *α*v*β*5,^41^ with Mer-dependent signaling necessary for subsequent internalization. To further probe the mechanistic role of Mer in AC recognition and engulfment, we have now examined macrophages that predominantly use a Mer-dependent AC phagocytosis mechanism.^17, 23^ We show that in these cells, which do not express TIM4, Mer has the capacity to serve a unique dual role in mediating both tethering of ACs to the macrophage surface as well as subsequent AC engulfment.

## Results

### Rapid binding of TAM ligands to ACs

**Figure 1:**
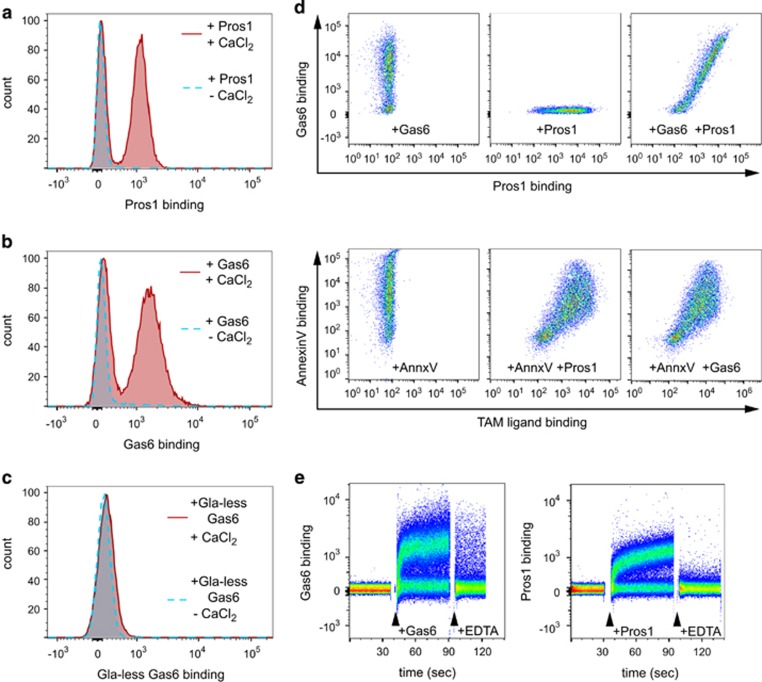
TAM ligand binding to apoptotic mouse thymocytes. (**a**–**c**) Binding of Dylight-488-labeled TAM ligands (Protein S: human Protein S, Gas6: full-length recombinant murine Gas6, Gas6-Gla: recombinant murine Gas6 lacking the N-terminal Gla domain) to Dex-treated thymocytes in the presence (solid red line) or absence (dotted blue line) of 1 mM CaCl_2_. (**d**) (Upper panels) Representative plots showing dual labeling of apoptotic PMN with Gas6 and Protein S, binding of Cy5-labeled Gas6 only (left) or Dylight-488-labeled Protein S only (center) or both labeled proteins together (right). (Lower panels) Representative plots showing labeling of apoptotic PMN with PE-labeled AnnexinV only (left) or with PE-labeled Annexin V, Cy5-labeled Gas6 and Dylight488-labeled Protein S. Plots for Annexin V and Protein S binding (center) and Annexin V and Gas6 binding (right) for the triple labeled cells are shown; data shown are representative of four separate experiments. (**e**) Representative plots of temporal flow cytometric analysis of Dylight-488 Gas6 and Protein S binding to apoptotic thymocytes. After ~30 s of data collection in the absence of ligand, Gas 6 (~15 nM final concentration) or Protein S (25 nM final concentration) was added and recording continued for ~45 s before addition of EDTA (final 5 mM) and recording continued for a further 25–30 s

Previous studies of TAM ligand binding to ACs have used indirect labeling techniques, including FITC-avidin to detect biotinylated ligand^28^ or labeled antibodies,^23, 42^ and these studies did not examine the kinetics of ligand binding. To examine directly the opsonization of ACs with TAM ligands, we used flow cytometric analysis of fluorescently labeled Protein S and Gas6. Labeled full-length Gas6 and Protein S were both functional, conferring TAM-dependent phagocytosis and capable of inducing phosphorylation of TAMs (data not shown). Importantly, fluorophore-labeled TAM ligands were found to bind to ACs (Figures 1a and b) in a manner consistent with data from indirect binding studies. Gla domain structure and PtdSer binding are known to be calcium-dependent^43^ and, accordingly, TAM ligands exhibited calcium-dependent binding to apoptotic thymocytes (Figures 1a and b). In contrast, recombinant Gas6 lacking the Gla domain did not bind to ACs even in the presence of calcium (Figure 1c). This observation is consistent with a requirement for the Gla domain for Gas6 binding to immobilized PtdSer^44^ and demonstrates that the Gla domain is required for Gas6 binding to ACs.

When apoptotic thymocytes were pre-incubated with 11 nM unlabeled Gas6 before incubation with labeled Protein S, the extent of subsequent Protein S binding was not reduced (mean fluorescence of Protein S binding in the absence of Gas6: 2003±236; in the presence of Gas6: 2110±380; unlabeled control: 55±9; mean±S.E.M., *n*=4). Similar results were obtained when ACs were pre-incubated with 100 nM unlabeled Protein S before addition of labeled Gas6 (data not shown). We therefore used multi-parameter flow cytometry to demonstrate that physiological concentrations of Gas6 and Protein S can simultaneously bind to Annexin V-positive ACs. For this series of experiments, we used human polymorphonuclear cells (PMN) that had been cultured *in vitro* for 20 h, which consistently induces ~70% apoptosis.^6^ In dual color labeling experiments, we observed that Cy5-labeled Gas6 (58 nM) and Dylight488-labeled Protein S (55 nM) bound to the same subpopulation of cells, with no reduction in binding when compared with single label only controls (Figure 1d, top panels). Next, we used three color flow cytometry to demonstrate that either pre- or co-incubation with the PtdSer binding protein Annexin V resulted in co-labeling of ACs with Gas6, Protein S, and Annexin V, whereas viable Annexin V-negative cells did not bind either Gas6 or Protein S (Figure 1d, lower panels). Importantly, binding of Annexin V did not reduce the binding of either Gas6 or Protein S when compared with single labeled cells: all of the Annexin V^+^ cell population in the lower panels of Figure 1d are shifted to the right following co-addition of Gas6 and Protein S (Figure 1d, lower panels). This observation indicates that at ~50 nM concentrations, the labeled TAM ligands neither saturate nor compete for available PtdSer sites expressed on the surface of ACs in this assay (see Discussion). This is the first demonstration that, in the simultaneous presence of physiological concentrations of Gas6 and Protein S, both ligands and other PtdSer-binding proteins can be co-bound to the AC surface.

Binding of TAM ligands to cells has previously been examined following relatively long incubation times, followed by washing and incubation with secondary detection agents.^28, 42^ In preliminary experiments, complete binding of labeled TAM ligands occurred following short incubations with ACs (<5 min, data not shown). We undertook a real-time flow cytometric analysis of labeled Gas6 and Protein S binding directly to ACs, without washing unbound ligand away (Figure 1e). Importantly, specific TAM ligand binding occurred within seconds, reaching near saturation levels of binding within a minute. Addition of 5 mM EDTA reversed binding immediately (Figure 1e), an effect that did not involve quenching of the fluorescence of labeled protein. Rapid reversal of binding of TAM ligands following the chelation of extracellular Ca^2+^ is consistent with Ca^2+^-dependent binding of TAM ligands to ACs (Figures 1a and b). Our demonstration of rapid and specific binding of either TAM ligands to AC targets suggests that even transient exposure would be sufficient to mark the AC for clearance by TAM-expressing phagocytes. Furthermore, the potential for ACs to be simultaneously opsonized with multiple PtdSer binding proteins under physiological conditions has significant implications for the control of AC removal at different tissue sites *in vivo*.

### Mer-dependent phagocytosis of Gas6-opsonized ACs

**Figure 2:**
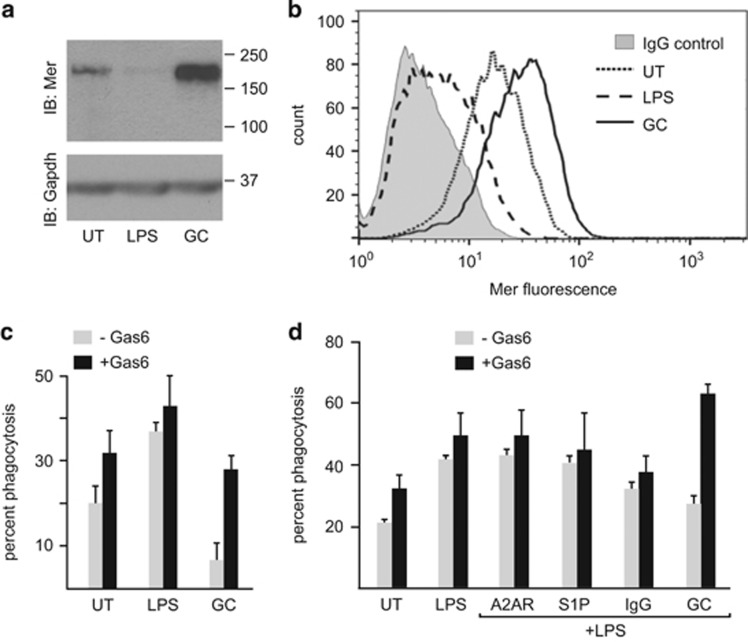
Differential regulation of macrophage Mer expression and TAM-dependent phagocytosis of ACs following treatment with LPS or GCs. (**a**) Determination of total levels of Mer expression in untreated (UT), LPS-treated (100 ng/ml), or GC-treated (Dex 100 nM) mouse BMDM. Macrophage lysates (10 μg total protein) were separated by SDS–PAGE, transferred to PVDF membranes, and probed with a Mer antibody and then stripped and re-probed for Gapdh. A representative immunoblot showing upregulation of Mer expression following GC treatment and downregulation of Mer in response to LPS. (**b**) Determination of cell surface expression of Mer in untreated (UT), LPS-treated (100 ng/ml), or GC-treated (Dex 100 nM) BMDM using indirect immunostaining and flow cytometry. A representative histogram overlay for gated macrophage populations is shown to illustrate upregulation of surface expression of Mer following GC treatment (solid line) and downregulation of Mer following LPS treatment (large dashed line). Binding of non-specific goat immunoglobulins is also shown (gray profile). (**c**) Mouse BMDM were treated for 20 h with LPS (100 ng/ml), GCs (Dex 100 nM), or untreated (UT). Macrophage phagocytosis of pHrodo-labeled apoptotic thymocytes performed without Gas6 (gray bars) or with Gas6 (black bars) was determined by flow cytometric assessment (mean percent phagocytosis±S.D. is shown, *n*=9), as described in the text. (**d**) Mouse BMDM were treated for 20 h with LPS together with different agonists that have been reported to suppress LPS effects; immune complexes (IgG; 40 μg/ml), sphingosine-1-phosphate (S1P; 1 μM), adenosine A2AR agonist CGS21680 (A2AR; 10 μM), or Dex (100 nM). Macrophage phagocytosis of pHrodo-labeled apoptotic thymocytes performed without Gas6 (gray bars) or with Gas6 (black bars) was determined by flow cytometric assessment (mean percent phagocytosis±S.D. is shown, *n*=5)

As reported previously,^17^ treatment of bone marrow-derived macrophages (BMDM) for 24 h with anti-inflammatory GCs (100 nM dexamethasone (Dex) for 24 h (GC-treated BMDM) resulted in increased total cellular expression of Mer as assessed by both immunoblot analysis (Figure 2a) and surface receptor expression by flow cytometric analysis (Figure 2b). In contrast, LPS-treated macrophages (100 ng/ml for 24 h) had very low levels of total and cell surface expression of Mer (Figures 2a and b). Gas6 serves as a ligand for all TAM receptors,^24, 37^ and so quantification of phagocytosis of either unopsonized or Gas6-opsonized AC targets allows for assessment of TAM-independent and TAM-dependent phagocytosis. Comparison of the capacity for TAM-independent phagocytosis (absence of Gas6) of ACs by macrophage populations revealed that GC-treated BMDM were relatively inefficient at phagocytosis of ACs when compared with LPS-treated BMDM (Figure 2c). However, in the presence of Gas6-opsonized ACs, GC-treated BMDM become very effective phagocytes, utilizing a predominantly TAM-dependent AC clearance mechanism (Figure 2c). In contrast, although LPS-treated BMDM exhibit a significant augmentation of AC phagocytosis when compared with untreated BMDM, this phagocytosis is predominantly Gas6-independent (Figure 2c).

We next examined the utilization of TAM receptors following combinatorial treatment of BMDM for 24 h with LPS and different agonists that, like GCs, have been reported to suppress pro-inflammatory cytokine production,^45, 46, 47^ including immune complexes (IgG; 40 *μ*g/ml), sphingosine-1-phosphate (S1P; 1 μM) and adenosine A2AR agonist (CGS21680; 10 μM). Combinatorial treatment of BMDM with LPS and A2AR agonists, S1P, or immune complexes did not affect the utilization of the largely TAM-independent AC phagocytosis mechanism when compared with LPS treatment alone (Figure 2d). In contrast, following combinatorial treatment with LPS and GCs, a marked TAM-dependent phagocytosis of ACs was observed (Figure 2d). Thus, even in a pro-inflammatory microenvironment GCs strongly promote utilization of a predominantly TAM-dependent AC clearance mechanism.

**Figure 3:**
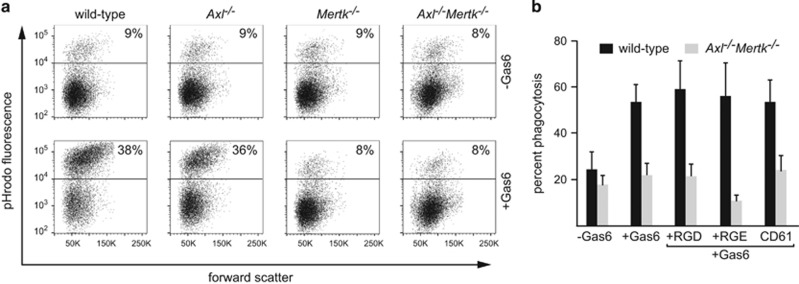
Mer-dependent phagocytosis of apoptotic cells by GC-treated macrophages. (**a**) Phagocytosis of pHrodo-labeled apoptotic thymocytes by mouse GC-treated BMDM was assessed by flow cytometry. Representative plots showing forward scatter v. pHrodo fluorescence for gated macrophage populations from wild type, *Axl*^*−/−*^, *Mertk*^*−/−*^, and *Axl*^*−/−*^*Mertk*^*−/−*^ mice in the absence (-Gas6) or presence (+Gas6) of 11 nM Gas6. (**b**) Phagocytosis of apoptotic thymocytes by GC-treated BMDM from either wild type (black bars) or *Axl*^*−/−*^*Mertk*^*−/−*^ (gray bars) mice was assessed by flow cytometry in either the absence, or the presence of Gas6 together with the addition of 1 mM GRGDSP (RGD), 1 mM GRGESP (RGE), or 10 μg/ml CD61 mAb. Results are presented as the percentage of pHrodo-positive (phagocytic) macrophages (mean±S.D., *n*=3)

In addition to Mer, Axl is expressed by BMDM cultures in a manner that is dependent on activation status.^17^ To definitively identify the contribution of Axl and Mer in the phagocytosis of ACs, we examined Gas6-dependent and independent AC phagocytosis by GC-treated BMDM derived from wild-type, *Mertk*^*−/−*^, *Axl*^*−/−*^ mice, and *Axl*^*−/−*^*/Mertk*^*−/−*^ double-knockout mice. The gross morphological appearance of BMDM and surface expression of F4/80 or CD11b was similar for all genotypes examined, suggesting that macrophage differentiation was not significantly affected by absence of TAMs (data not shown). GC-treated BMDM from both wild-type and *Axl*^*−/−*^ mice exhibited significant, and similar, Gas6-dependent phagocytosis of ACs (Figure 3a). The lack of any effect due to *Axl* gene deletion is consistent with the fact that GC-treated BMDM express abundant Mer (Figure 2a), but no little or no Axl.^17^ In contrast, GC-treated BMDM prepared from *Mertk*^*−/−*^ or *Axl*^*−/−*^*/Mertk*^*−/−*^ mice did not display any increase in phagocytosis of ACs on addition of Gas6 (Figure 3a). Therefore, GC-treated BMDM constitute a model in which the bulk of AC phagocytosis is Mer-dependent.

### Integrin-independent AC tethering

Previous studies in other phagocyte populations have suggested that Mer-dependent phagocytosis requires integrin-mediated tethering of ACs.^41^ We therefore assessed the effects of pre-treatment of mouse GC-treated BMDM with either GRGDSP peptides or an integrin β3 (CD61) blocking monoclonal antibody (mAb) on Gas6-stimulated phagocytosis. We did not observe inhibition of such phagocytosis by either treatment (Figure 3b). The function-blocking *β*3 integrin antibody could be shown to bind to GC-treated BMDM by flow cytometric analysis, and the activity of the GRGDSP peptide was confirmed by detachment of fibroblasts from fibronectin-coated substrates (data not shown). We further tested whether phagocytosis of ACs by human monocyte-derived GC-treated macrophages could be affected by pre-treatment with blocking antibodies against either *β*3 integrin or *β*5 integrin. Pre-treatment of GC macrophages with either mAb also failed to reduce AC phagocytosis in the presence of Protein S (control mAb: 68±4% *β*5 mAb: 65±6%, β3 mAb: 67± 8%, mean±S.D., *n*=3). Together, these data raised the possibility that phagocytosis of ACs by GC-treated macrophages was independent of *α*V integrin-mediated tethering.

### Mer-dependent tethering of ACs to GC-treated macrophages

**Figure 4:**
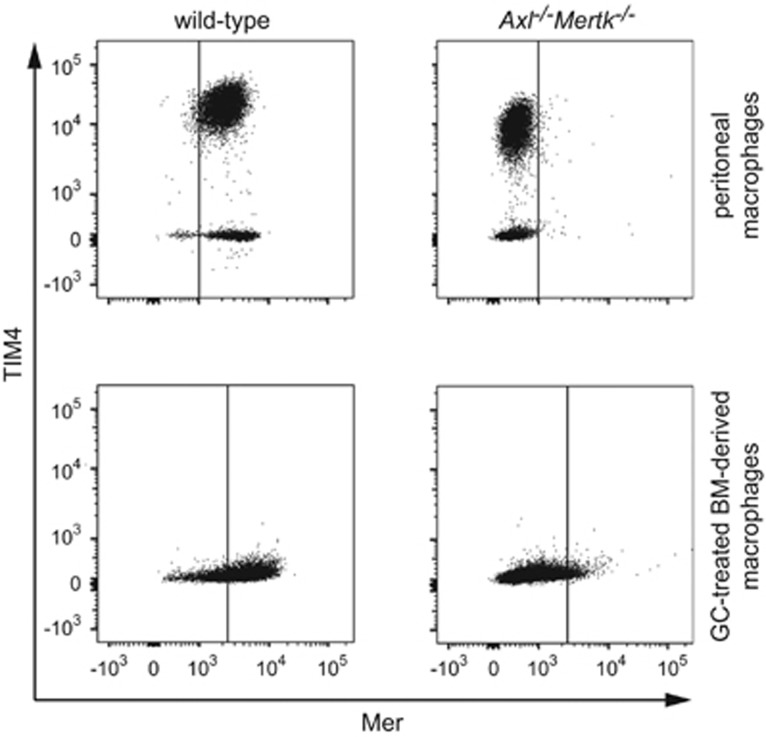
Differential expression of TIM4 on peritoneal and GC-treated macrophages. Expression of TIM4 and Mer on mouse macrophages was determined by two-color immunofluorescence analysis. Macrophage populations from Axl/Mer ^*−/−*^ animals that had been labeled with a Mer antibody were used to define the threshold for positive staining for Mer, with <1% positive cells. The same threshold value was then used for equivalent macrophage populations from wild-type animals. Upper panels: peritoneal cells were collected from untreated mice, and macrophages were gated on the basis of forward scatter and side scatter. Representative two-color plots for the expression of Mer and TIM4 on gated macrophage populations from wild type and *Axl*^*−/−*^*Mertk*^*−/−*^ animals, clearly defining two distinct populations: Mer+/TIM4+ macrophages (~80% of the cells) and Mer+/TIM4− macrophages (~20% of cells) (upper left panel). The proportions of TIM4+ and TIM4− macrophages is unchanged in peritoneal macrophages obtained from Axl/Mer^*−/−*^ animals (upper right panel). Lower panels: mouse GC-treated BMDM were analyzed for expression of Mer and TIM4. Representative two-color histograms show expression of Mer and TIM4 on GC-treated macrophages from wild type and *Axl*^*−/−*^*Mertk*^*−/−*^ mice, demonstrating that mouse BMDM lack TIM4 (lower left panel)

In view of a recent report indicating that TIM4 acts co-operatively with Mer to mediate tethering of ACs to peritoneal macrophages,^39^ we next considered the possibility that TIM4 was required for phagocytosis of ACs by GC-treated BMDM. However, we found that unlike peritoneal macrophages, BMDM do not express TIM4. Mer is strongly expressed by peritoneal macrophages, and a major subpopulation of these cells expresses TIM4 (Figure 4, upper left panel). Expression of TIM4 on this peritoneal macrophage population was not affected by the absence of expression of Axl and Mer (Figure 4, upper right panel). Although GC-treated BMDM express Mer (Figures 2a and b, and 4, lower left panel), they do not express TIM4 (Figure 4, lower panels), and so this protein cannot have a role in the tethering of ACs to these cells.

**Figure 5:**
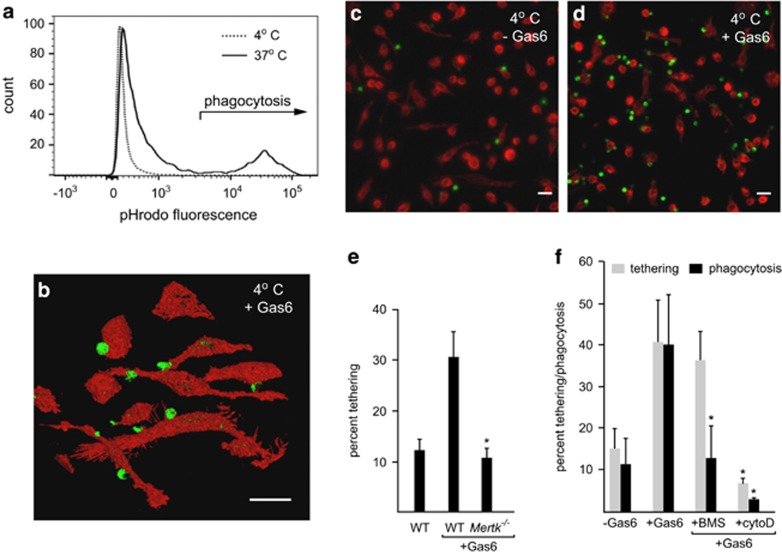
Tethering of apoptotic thymocytes to murine GC-treated macrophages. (**a**) Flow cytometric analysis of the capacity for mouse GC-treated BMDM phagocytosis of apoptotic thymocytes at either 37 or 4 °C. Apoptotic thymocytes (pHrodo-labeled) were co-cultured with mouse GC-treated BMDM for 30 min before detachment with trypsin-EDTA and analysis of fluorescence associated with gated macrophage populations. A typical histogram overlay showing macrophage fluorescence following co-incubation with labeled thymocytes at 37 °C (solid line) *versus* 4 °C (dotted line). The fluorescence intensity typically associated with phagocytosis of apoptotic cells is highlighted. (**b**) Representative confocal microscopy image showing 45° angle projected composite of serial Z-series images of apoptotic thymocyte (green) tethering to mouse GC-treated BMDM (red) at 4 °C. Scale bar= 20 μm. (**c**, **d**) Tethering of apoptotic targets to mouse GC-treated BMDM. Representative micrographs of tethering of CMFDA-labeled apoptotic thymocytes (green) to phalloidin-labeled GC-treated BMDM (red) at 4 °C for 30 min in the absence (**c**) and presence (**d**) of 11 nM Gas6. Scale bar=20 μm. (**e**) GC-treated BMDM from either wild type or *Mertk*^*−/−*^ mice were co-incubated with apoptotic thymocytes at 4 °C in the absence or presence of 11 nM Gas6. Tethering was quantified by microscopy analysis and the mean percentage of BMDM with tethered ACs was determined, *significant difference (*P*<0.05) from WT+Gas6 (mean±S.D., *n*=5). Gas6-dependent tethering of apoptotic thymocytes that is observed for wild type GC-treated BMDM is not observed for BMDM derived from *Mertk*^*−/−*^ mice. (**f**) The effect of pre-treatment of mouse GC-treated BMDM either with BMS777607 (BMS) to block Mer tyrosine kinase activity or with cytochalasin D (CytoD) to disrupt cytoskeletal integrity, on tethering (grey bars) or phagocytosis (black bars) of apoptotic thymocytes. The percentage of BMDM tethering or phagocytosis was quantified by either microscopy (mean±S.D., *n*=4) or flow cytometry (*n*=3) respectively. *Indicates significant difference (*P*<0.05) from Gas6 alone

We therefore established an assay to measure tethering of ACs to the macrophage surface to identify the receptors involved in this function. We observed significant tethering of ACs to GC-treated BMDM following co-incubation of ACs at 4 °C, a temperature at which membrane internalization is inhibited. We confirmed that under these conditions, GC-treated BMDM failed to internalize labeled ACs (<2% internalization), demonstrated by flow cytometric analysis of macrophages following treatment with trypsin/EDTA to remove tethered ACs (Figure 5a). Tethering, as opposed to internalization, of ACs to GC-treated BMDM was further confirmed by laser scanning confocal microscopy analysis (Figure 5b). Using the microscopy-based assay we observed that tethering of ACs to GC-treated BMDM was TAM ligand-dependent (Figures 5c and d). This suggested that in the GC-treated BMDM Mer acts as a tethering receptor. Indeed, macrophage expression of Mer was required for the AC tethering, as GC-treated BMDM from *Mertk*^*−/−*^ animals failed to show Gas6-dependent tethering of ACs (Figure 5e).

### Tethering and engulfment differ in their requirement for TAM kinase activity

We expected that since Mer tyrosine kinase activity is significantly inhibited at 4 °C and since tethering is observed at this temperature, kinase activity would not be required for Mer-dependent AC tethering. To test this, we examined the effects of the TAM kinase inhibitor BMS777607, which we have previously demonstrated is a potent inhibitor of TAM ligand-induced Axl and Mer phosphorylation.^17, 48, 49^ Although TAM ligand-dependent phagocytosis of ACs was potently blocked by pre-incubation of GC-treated macrophages with BMS777607,^17^ AC tethering was not affected (Figure 5f). These data demonstrate that tethering is independent of Mer tyrosine kinase activity, but that Mer-dependent signaling is required for AC internalization by macrophages. In a series of experiments to examine the effects of cytoskeletal disruption using cytochalasin D on tethering and phagocytosis, we observed that pre-treatment of GC-treated macrophages with cytochalasin D completely inhibited both tethering and phagocytosis of ACs (Figure 5f). These data suggest that the actin cytoskeleton is required for both Mer-dependent tethering and subsequent phagocytosis.

### Mer is a tethering receptor in human GC-treated macrophages

**Figure 6:**
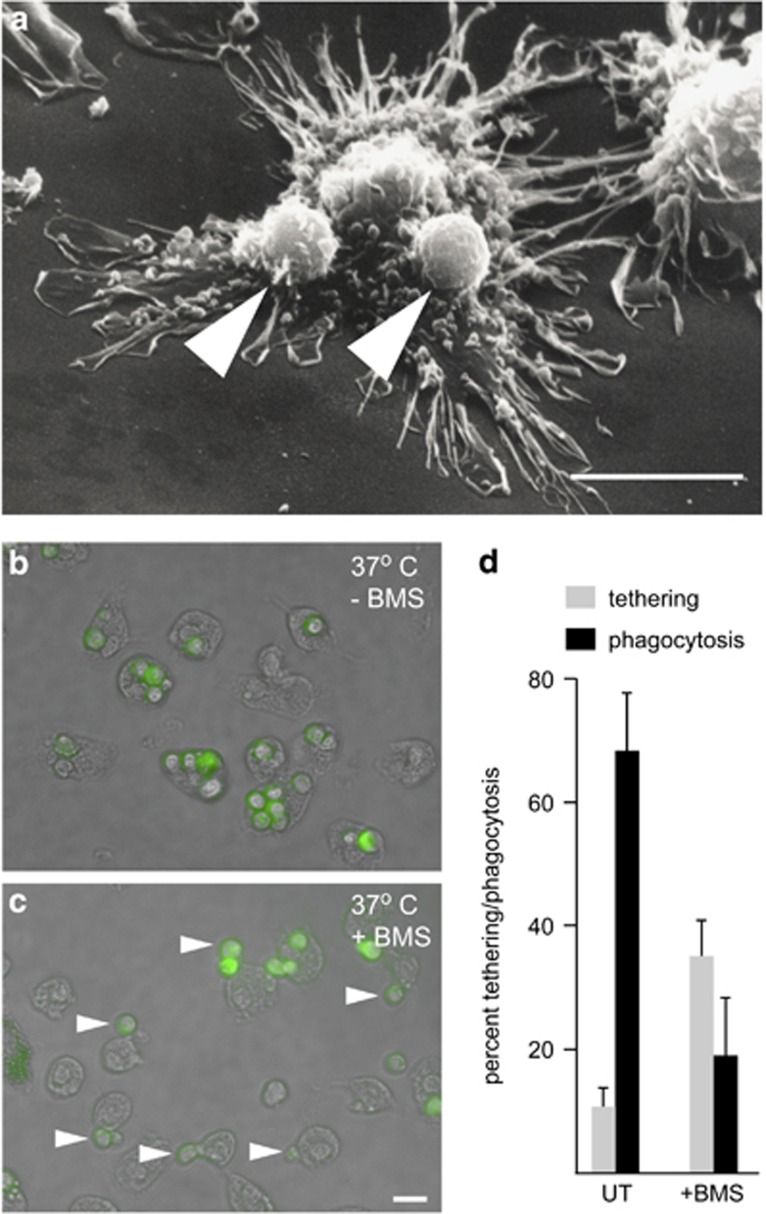
Mer-dependent tethering of apoptotic thymocytes to human GC-treated macrophages. (**a**) Scanning electron micrograph of apoptotic human PMN (arrowed) tethering to human monocyte-derived GC-treated macrophages under equivalent conditions to those used for mouse GC-treated macrophages and apoptotic thymocytes shown in Figure 5b and d. Scale bar=10 μm. (**b**, **c**) Merged fluorescent/phase contrast images of human monocyte-derived GC-treated macrophages incubated with apoptotic human PMN (labeled with CMFDA—green) in the presence of 35 nM Protein S at 37 °C for 30 min in the absence (**b**) or presence (**c**) of 300 nM BMS777607. Arrows highlight tethering of labeled apoptotic PMN to human GC-treated macrophages. Scale bar= 20 μm. (**d**) Quantification of tethering/phagocytosis of apoptotic PMN by human monocyte-derived GC-treated macrophages following co-incubation at 37 °C in the presence of 35 nM Protein S for 30 min. GC-treated macrophages were either DMSO-treated (UT, untreated) or pre-treated with 300 nM BMS777607. The proportion of GC-treated macrophages, which showed tethering (gray bars, see Figure 6c) *versus* phagocytosis (black bars, see Figure 6b), was determined by microscopy analysis with quantification of at least 500 cells in 5–7 randomly selected fields using a x40 objective. Data shown are mean±S.D., *n*=4

**Figure 7:**
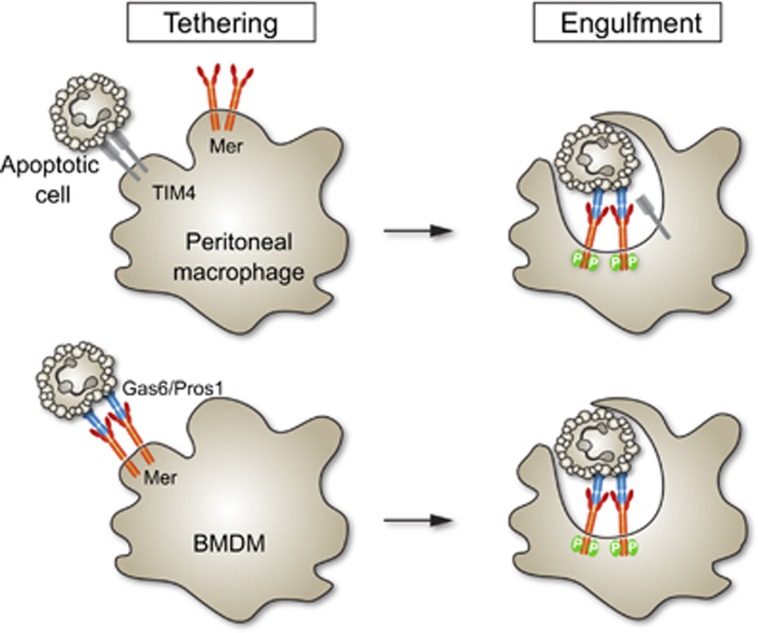
Schematic of Mer-mediated tethering and phagocytosis of apoptotic cells (ACs) in peritoneal *versus* bone-marrow derived macrophages. The former use TIM4 for AC tethering and Mer for AC phagocytosis,^39^ whereas the latter use Mer for both. Tethering does not require Mer kinase activity, whereas phagocytosis does

Tethering of ACs could be also observed for human GC-treated macrophages incubated with ACs at 4 °C, as revealed by scanning electron microscopy (Figure 6a, arrows). Therefore, we used human monocyte-derived GC-treated macrophages (which exhibit a higher percentage of TAM-dependent phagocytosis when compared with mouse GC-treated BMDM), to test the prediction that inhibition of Mer kinase activity with BMS777607 in human GC macrophages should result in increased AC tethering. Inhibition of AC phagocytosis following pre-incubation of human GC-treated macrophages with BMS777607 was confirmed using a flow cytometric assay (phagocytosis in the absence of Protein S; 24+2.6%, Protein S alone; 69+9%, Protein S+BMS777607; 35.7+7.7%, *n*=4 *P*<0.05), consistent with data from mouse GC-treated BMDM (Figure 5f). To detect tethering macrophage monolayers were washed multiple times before quantification of phagocytosis and tethering by microscopy. For GC-treated macrophages in the absence of BMS777607, the vast majority of macrophages had internalized ACs during the 30-min assay, with a small percentage of tethered ACs (Figure 6b). In contrast, GC-treated macrophages incubated with BMS777607 showed increased tethering of ACs (Figure 6c). Quantification of AC tethering *versus* internalization revealed that inhibition of Mer tyrosine kinase activity resulted in a threefold increase in the percentage of macrophages with tethered ACs (Figure 6d), with a concomitant reduction in the percentage of internalized ACs (Figure 6d). These data demonstrate that in macrophages that lack TIM4, Mer can uniquely serve as a receptor that is required and capable of mediating both tethering and the subsequent phagocytosis of AC (Figure 7). Mer tyrosine kinase activity is required for the latter, but not for the former.

## Discussion

We have identified a novel dual role for the Mer RTK in both the capture (tethering) and subsequent internalization of ACs. Both of these events require the binding of the Gla domain of a TAM ligand, either Gas6 or Protein S, to PtdSer expressed on the surface of ACs. Our data demonstrate that this binding is rapid and Ca^2+^-dependent, and is rapidly reversed when Ca^2+^ is removed. Biochemical analyses have revealed the presence of multimeric forms of Protein S in plasma,^50^ and previous studies have suggested that Protein S may also undergo oligomerization following binding to phospholipids in membranes, an event that requires disulfide bond formation.^51^ Our data indicate that any AC membrane-induced oligomerization of Protein S does not prevent subsequent dissociation of the ligand on chelation of extracellular calcium.

The simultaneous AC co-binding by physiological concentrations of TAM ligands and Annexin V that we document has important implications for potential co-opsonization of ACs with multiple PtdSer ligands. Occupancy of all PtdSer binding sites with ligand requires exceptionally high concentrations of PtdSer opsonins.^28^ At physiological concentrations of Protein S and Gas6, we found that apoptotic thymocytes were able to bind both ligands simultaneously. One prediction is that under normal physiological conditions, for ACs that have bound TAM ligands, additional (unoccupied) PtdSer sites could be recognized by other PtdSer binding proteins such as MFG-E8^20^ or C1q^52^ and could engage multiple phagocyte clearance receptors such as TIM4 and BAI1.

Our previously published work,^17, 23^ together with the new data presented here, unequivocally demonstrate that Mer is required for TAM ligand-dependent phagocytosis of ACs by GC-treated BMDM. Axl is not expressed by these cells,^17^ and therefore Mer carries the full burden. Although *β*3 and *β*5 integrins are expressed by GC-treated macrophages, the lack of inhibition of TAM-dependent phagocytosis following treatment with RGD containing peptides or function-blocking antibodies suggests that integrins are not required for AC phagocytosis by these cells. One of the assays that we have used further excludes a role for integrins in the tethering of ACs by GC-treated macrophages, as integrins are likely to be inactive at 4 °C. In addition, the tethering we observe was found to be optimal in the presence of Ca^2+^, not Mn^2+^.^53^ Our findings demonstrate that the two-way communication between *α*V integrin and Mer that has been reported in RPE cells^54^ does not have a significant role in GC-treated macrophages.

Peritoneal macrophages express unusually high levels of TIM4 and are able to tether apoptotic targets via TIM4 without a requirement for Mer,^39, 55^ but we show that GC-treated BMDM do not express TIM4 and therefore cannot tether ACs with this receptor. Although peritoneal macrophages are widely used for functional analyses, they represent an unusual population of cells associated with immunity at serosal surfaces.^56^ In contrast, there are many phagocytic tissue macrophage populations *in vivo*, for example, the microglia of the central nervous system, that are, like GC-treated BMDM, strongly Mer-positive but TIM4-negative.^57^ TIM4 cannot act as a PtdSer receptor in these macrophages. Recent transcriptome-based analyses reveal that GC-treated macrophages exhibit considerable overlap with those treated with IL-10, highlighting the spectrum of macrophage phenotypes that can be identified beyond a simple M1/M2 dichotomy.^58^ Undoubtedly, the molecular cooperativity required for the tethering and subsequent clearance of ACs will be different for different phagocytes.

An interesting recent article suggests that Axl mediates tethering of AC to dendritic cells,^59^ supporting the idea that, in addition to conveying signals that promote internalization, TAM receptors have an important role in the capture of apoptotic targets by phagocytes. However, internalization of ACs tethered via Axl was suggested to require formation of a molecular complex of Axl and LDL receptor-related protein-1,^59^ raising the possibility that the mechanism by which Mer and Axl mediate tethering and internalization of ACs is distinct. Our data demonstrate that Mer is able to function as a fully competent AC tethering receptor in GC-treated macrophages, and can serve the additional function of signaling apoptotic cell internalization in a TAM tyrosine kinase-dependent manner.

Our results have important implications for the use of TAM receptor inhibitors, TAM ligands, or antibodies to target TAMs *in vivo*, as these different strategies for perturbing TAM function will exert distinct effects on AC tethering and phagocytosis. Use of small molecule inhibitors of TAM kinases (such as BMS777607) would allow AC tethering, but prevent downstream signaling necessary for phagocytosis and TAM-mediated suppression of pro-inflammatory cytokine production.^17^ In contrast, some Mer antibodies would be predicted to block AC tethering and phagocytosis by binding directly to receptor sites involved in TAM ligand recognition. Certain of these ligand-blocking Mer antibodies can nonetheless exert agonistic effects on Mer tyrosine kinase activity, presumably through their ability to crosslink the receptor.^17^ Thus, TAM-dependent anti-inflammatory signaling could be preserved in the absence of AC tethering/phagocytosis. The high levels of PtdSer exposure on ACs may make it difficult to achieve effective blockade of AC opsonization with TAM ligands, despite the extremely rapid binding to exposed PtdSer. However, we have recently demonstrated that Gla-less Gas6 retains the capacity to bind to TAM receptors, but is seriously compromised with respect to activation of TAM kinase activity.^24^ Thus, Gla-less TAM ligands may competitively inhibit TAM-dependent AC tethering and differ from antibodies and small-molecule TAM kinase inhibitors in their mode of action. Our observations may facilitate new approaches to therapeutic modulation of Mer function either in homeostasis or in disease. Separation of the contribution of these two processes is important, as *in vitro* studies have demonstrated that interaction of macrophages with ACs without engulfment can confer AC-induced anti-inflammatory effects.^60^ Importantly, although Mer kinase activity is not necessary for tethering, the Mer tyrosine kinase is nonetheless probably activated at the tethering stage.

In summary, we show that Mer has dual activities and can function as a mediator of both AC tethering and subsequent phagocytosis. Our definition of the molecular mechanisms underlying phagocyte-AC interactions may provide opportunities to identify novel targets for the restoration of defective AC clearance associated with the development of inflammatory disease and autoimmunity.

## Materials and Methods

### Reagents

Reagents were obtained from Sigma-Aldrich (St. Louis, MO, USA) unless otherwise stated. DMEM and Iscove's DMEM (IDMEM) were from Invitrogen (Carlsbad, CA, USA). Human Protein S was obtained from Enzyme Research Laboratories (South Bend, IN, USA). Recombinant mouse Gas6 (full-length and Gla-less) was prepared as previously described.^24, 48^ BMS777607 was obtained from Selleckchem (Houston, TX, USA). Goat polyclonal antibodies against human Mer and mouse Mer and Axl and were obtained from R&D Systems (Minneapolis, MN, USA). Monoclonal anti-human Mer (mouse IgG1) was from R&D Systems, anti-GAPDH (mouse IgG1) was from Millipore (Billerica, MA, USA), anti-mouse CD61 mAb (Hamster, Hmbeta 3.1) and TIM4 (Rat IgG2a – clone 54) were obtained from eBioscience (San Diego, CA, USA). Control immunoglobulins, and secondary HRP or fluorescent-conjugated antibodies were from DAKO (Carpinteria, CA, USA). All antibodies were titrated on cells or cell lines known to express the antigen. Enhanced chemiluminescence (ECL) substrate and Hyperfilm was obtained from GE Healthcare (Pittsburgh, PA, USA).

### Cell isolation and culture

Human blood monocytes and PMN leukocytes were isolated and cultured as described.^23^ Ethical approval for blood cell isolation was obtained from the Lothian Research Ethics Committee (#08/S1103/38 or #1702/95/4/72) at the Queen's Medical Research Institute, University of Edinburgh. All healthy volunteers provided informed written consent. In brief, erythrocytes were sedimented from freshly drawn peripheral blood with 0.6% (w/v) dextran T500 followed by fractionation of leukocytes on a discontinuous Percoll gradient (prepared in Ca^2+^/Mg^2+^-free phPBS with final concentrations of Percoll of 50, 63, and 73%) at 720 g for 20 min. Mononuclear cells (MNC) were aspirated from the 50/63 interface, and PMN from the 63/73% interface, and washed three times in PBS (without Ca^2+^/Mg^2+^) before culture. Autologous platelet-rich plasma-derived ‘serum' was obtained by addition of CaCl_2_ (20 mM final) to platelet-rich plasma and incubation in a glass tube for 1 h at 37 °C. MNC were plated at 4 × 10^6^/ml in IDMEM and incubated for 45–60 min, at 37 °C, 5% CO2 after which non adherent lymphocytes removed by washing with HBSS (without Ca^2+^/Mg^2+^). Alternatively, monocytes were isolated by negative selection using monocyte isolation kit II as specified by the manufacturer (Miltenyi Biotech, Bisley, Surrey, UK) and plated at 6 × 10^5^ cells/ml. Monocytes were cultured for a period of 5–7 days in IDMEM containing 10% autologous serum, with or without the addition of 250 nM (Dex) as detailed in the figure legends, PMN (resuspended at 4x10^6^ cells/ml in IDMEM containing 1% BSA) were cultured at 37 °C in a 5% CO2 atmosphere for 20 h in Falcon tissue culture flasks (BD Biosciences, San Jose, CA, USA). Cultured PMN populations were routinely >60% apoptotic, as determined by morphological analysis and Annexin V binding, and <5% propidium iodide positive. Mouse BMDM were prepared from wild-type C57bl/6 or TAM-knockout mice^35^ bred and housed at the Salk Institute Animal Facility and cultured for 6–9 days as described.^17, 61^ The *Mertk*^*−/−*^ mice used in this study are the Mer kinase-dead mutants reported by Camenisch *et al.*^62^ Although these mice express a low level of a truncated mRNA,^35^ the truncated Mer protein is not stable and they are therefore effectively complete Mer protein knockouts.^33, 63^ In brief, tibias and femurs from 6- to 8-week-old mice were flushed with sterile PBS (Mediatech Corning, Manassas, VA, USA), and red blood cells were lysed with ACK lysis buffer using the manufacturer's protocol (Lonza, Walkersville, MD, USA). Bone marrow cells were plated on Petri dishes in DMEM supplemented with 10% FBS, antibiotics and 20% L929 supernatant as a source of macrophage colony-stimulating factor. Fresh differentiation media was added on day 4 and macrophages were used on day 7–8. For treatment with glucocorticoids, macrophages were incubated for 24 h with Dex (100 nM). In some experiments, macrophages were treated for 24 h with LPS (100 ng/ml) or LPS in combination with sphingosine-1-phosphate (1 μM), adenosine A2AR agonist (CGS21680, 10 μM), immune complexes (biotinylated BSA mixed with mouse anti-biotin IgG1, 40 μg/ml final IgG concentration), or 100 nM Dex. Mouse thymocytes were isolated by mechanically disrupting the thymus with 19-G needles followed by lysis of red blood cells with ACK lysis buffer. Thymocytes were cultured for 6 h in DMEM/10% FCS containing 5 × 10-7M Dex to induce apoptosis (64±8.2% Annexin V+, *n*= 11).

### Macrophage tethering and phagocytosis of ACs

Macrophage phagocytosis of labeled ACs was assessed by flow cytometry as described,^17, 64, 65^ using cells that had been washed extensively to remove all possible exogenous TAM ligands or soluble TAM receptors. BMDM (mouse) or monocyte-derived macrophages from blood (human) were overlaid with ACs that had been labeled either with 5-chloromethylfluorescein diacetate (CMFDA) or pHrodo. After co-culture for 30 min at 37 °C, non-ingested ACs were removed by aspiration of media and cells detached by addition of 0.25% trypsin containing 1 mM EDTA for 5 min at 37 °C followed by vigorous pipetting. The percentage of macrophages that were fluorescent was then determined by flow cytometric analysis as described below. For assessment of AC tethering, macrophages were overlaid with CMFDA-labeled ACs (phagocyte:target ratio of 1 : 5) and incubated at 4 °C for 30–60 min. Macrophage monolayers were washed three times in DMEM, fixed with 2.5% paraformaldehyde and counter-stained with rhodamine phalloidin. For quantification of tethering, we used a modification of a well-characterized microscopy-based assay for phagocytosis.^66^ If an AC target was observed to be within the membrane of a macrophage, it was recorded as being internalized, whereas AC tethering was identified by the presence of ACs directly adjacent to macrophage membrane. This method of quantification was validated by confirmation that tethered AC targets remained accessible to an antibody, whereas internalized ACs were not (data not shown). For experiments using the inhibitor BMS777607, a similar analysis was undertaken to assess the effect of BMS777607 on particle tethering or internalization. In these experiments, macrophages were washed once then pre-incubated for 15 min with 300 nM BMS777607 before co-incubation with AC targets at either 4 or 37 °C. The percentage of macrophages associated with targets was quantified microscopically (>500 cells in randomly selected fields per well) and an average between duplicate wells calculated.

### Labeling of proteins

Proteins were labeled with DyLight-488 N-hydroxysuccinimide ester as recommended by the manufacturer (Pierce, Rockford, IL, USA). In brief, protein to be labeled (at 1.0–1.2 mg/ml) was exchanged into 100 mM boric acid, 25 mM sodium borate and 70 mM NaCl (pH8.4) and incubated with a 10-fold molar excess of DyLight-488 (stock prepared in dimethylformamide). After labeling for 30–60 min in the dark at room temperature, the reaction was terminated by addition of glycine to a final concentration of 50 mM and then protein was buffer exchanged into PBS. The degree of protein labeling was estimated from measurement of absorbance at 280 and 493 nm and was routinely between 2.5 and 3.5 moles of dye/mole of protein. Alternatively, proteins were labeled with Cy5, exchanging protein to be labeled (at 1.0–1.2 mg/ml) into 100 mM sodium hydrogen carbonate buffer (pH8.8) and incubated with Cy5 mono-reactive dye pack for 30 min in the dark at room temperature as recommended by the manufacturer (GE Healthcare). After termination of the reaction by addition of glycine to a final concentration of 50 mM, protein was buffer exchanged into PBS using protein desalting spin columns (Pierce). The degree of protein labeling was estimated from measurement of absorbance at 280 and 493 nm (Dylight 488) or 280 and 650 nm (Cy5) and was routinely found to be between 2.5 and 3.5 moles of dye/mole of protein. Functionality of labeled protein was assessed by testing the potential to induce Mer phosphorylation and the capacity to confer Mer-dependent phagocytosis of ACs (data not shown).

### Flow cytometry

Indirect immunofluorescence analysis was performed as described^67^ with all incubations carried out on ice to prevent internalization of bound antibody. Macrophages were detached from tissue culture plastic by incubation in PBS containing 2 mM EDTA and 0.5% serum for 10–20 min. After washing with ice-cold PBS containing 0.2% (w/v) bovine serum albumin and 0.1% (w/v) sodium azide (PBN), cells (10^5^/assay) were labeled following blocking of non-specific binding of antibodies to Fc with 20% (v/v) normal rabbit serum or FcR block (eBioscience). Cells were then incubated with saturating concentrations of goat anti-mouse Mer antibody for 30 min and washed twice in PBN before incubation with FITC- or APC-conjugated anti-goat immunoglobulin (DAKO) for 30 min and washed twice more before analysis. For some experiments, macrophages were further labeled with PE-conjugated anti-TIM-4 or appropriate rat IgG2b isotype control. For TAM ligand binding, apoptotic mouse thymocytes or apoptotic human neutrophils were incubated with saturating concentrations of fluorescently labeled TAM ligands diluted in 20 mM HEPES pH7.4 containing 0.14 M NaCl with or without the addition of 2 mM CaCl_2_ on ice. For some experiments, apoptotic cells were co-incubated with PE-conjugated Annexin V. Data acquisition was carried out using an LSRII or FACSCalibur flow cytometer (BD Biosciences) with post-acquisition data analysis with either Cellquest (BD Biosciences) or Flowjo software (Flowjo, Ashland, OR, USA).

### Scanning electron microscopy

Monocytes were cultured adherent to clean glass coverslips in DMEM containing 10% autologous serum and 250 nM Dex for 5 days before washing in PBS and fixation in 3% glutaraldehyde in sodium cacodylate buffer (0.1 M, pH 7.4) for 3 h. Coverslips were washed three times in sodium cacodylate buffer (0.1 M, pH 7.4) for 20 min each and transferred to osmium tetroxide (1% v v-1) in sodium cacodylate buffer (0.1 M, pH 7.4) for 1 h. Following a 30-min wash in distilled water, samples were dehydrated by sequential washes in increasing concentrations of acetone for 1 h each (50, 70, 90%), followed by three washes in acetone (100%) for 1 h each. Critical point drying with carbon dioxide was carried out and samples had a sputter coating of gold:palladium (20 nm, 60/40) added before viewing with a Philips 505 scanning electron microscope.

### Western blot analysis

Cells were lysed for 15 min on ice in PBS containing 1% NP-40, 0.1 mM NaVO_3_, and protease inhibitor cocktail (Roche, Mannheim, Germany) before centrifugation at 14 000  × *g*, 4 °C, 20 min. The resulting lysates were tested for protein concentration using a detergent compatible protein estimation kit (Pierce) and equilibrated to contain equivalent levels of protein (10–15 μg total protein) and resolved using a 9% reducing SDS–PAGE. Proteins were transferred to PVDF membranes, which were then blocked with TBS containing 0.05% Tween-20 and 5% skim milk (w/v) before probing with antibodies. Bound antibodies were visualized with enhanced chemiluminescence as described by the manufacturer.

### Statistics

Results are presented as mean±S.D. where *n*=number of independent experiments using cells obtained from different donors or mice and significance analyzed by ANOVA with Mann–Whitney post-test using Instat software (Graphpad, San Diego, CA, USA).

## Abbreviations

ACs: apoptotic cells
BMDM: bone marrow-derived macrophage
GCs: glucocorticoids
Dex: dexamethasone
TAM: Tyro3/Axl/Mer
PtdSer: phosphatidylserine
RTK: receptor tyrosine kinase
RPE: retinal pigment epithelial
PMN: polymorphonuclear cells
LXR: liver X receptor
IL: interleukin
TIM4: T-cell immunoglobulin and mucin domain-containing molecule 4
Ca: calcium
LPS: lipopolysaccharide
